# Fabrication and photocatalytic properties of silicon nanowires by metal-assisted chemical etching: effect of H_2_O_2_ concentration

**DOI:** 10.1186/1556-276X-7-663

**Published:** 2012-12-05

**Authors:** Yousong Liu, Guangbin Ji, Junyi Wang, Xuanqi Liang, Zewen Zuo, Yi Shi

**Affiliations:** 1 College of Materials Science and Technology, Nanjing University of Aeronautics and Astronautics, Nanjing, 211100, People's Republic of China; 2 College of Physics and Electronics Information, Anhui Normal University, Wuhu, 241000, People's Republic of China; 3 College of Electronic Science and Engineering, Nanjing University, Nanjing, 210093, People's Republic of China

**Keywords:** Silicon nanowire arrays, H_2_O_2_, Photocatalytic properties

## Abstract

In the current study, monocrystalline silicon nanowire arrays (SiNWs) were prepared through a metal-assisted chemical etching method of silicon wafers in an etching solution composed of HF and H_2_O_2_. Photoelectric properties of the monocrystalline SiNWs are improved greatly with the formation of the nanostructure on the silicon wafers. By controlling the hydrogen peroxide concentration in the etching solution, SiNWs with different morphologies and surface characteristics are obtained. A reasonable mechanism of the etching process was proposed. Photocatalytic experiment shows that SiNWs prepared by 20% H_2_O_2_ etching solution exhibit the best activity in the decomposition of the target organic pollutant, Rhodamine B (RhB), under Xe arc lamp irradiation for its appropriate Si nanowire density with the effect of Si content and contact area of photocatalyst and RhB optimized.

## Background

Photocatalysis has attracted much interest due to its potential advantages in utilizing solar energy to degrade organic pollutants and develop new energy [1-4]. As a traditional photocatalyst, semiconductor TiO_2_ has enormous potential in photocatalysis, but its wide band gap (3.2 eV) limits the use of light energy [5,6].

Silicon materials, which exhibit a wide optical adsorption range, high optical absorption efficiency, and high electron mobility, become a great potential photoelectric conversion material for its important applications in the field of photovoltaics and photocatalysis [7-10]. The realization of the silicon structure, especially the preparation of nanowire arrays, is very significant for the development and production of efficient quantum devices, photoelectric devices, and electronic and optical sensors [11-15]. Various methods have been developed to prepare one-dimensional silicon nanostructures, such as chemical vapor deposition [16], supercritical fluid-liquid–solid synthesis [17], laser ablation [18], thermal evaporation decomposition [19], and other processes.

In recent years, a simple catalytic etching technique with metal particles as catalyst to prepare large-area aligned monocrystalline silicon nanowire arrays on silicon wafers has been reported [20-27]. The technique is actually a wet chemical corrosion, the process of which is relatively simple, low cost, and controllable. Recent works on the etching method with depositions of two-dimensional (2-D) micro/nanoparticle arrays [28-33] or 2-D nanopattern fabrications [34,35] with highly ordered configurations, which are applicable for enabling highly dense nanowire formation, have also been reported. The controlled depositions of micro/nanoparticles result in close-packed highly ordered 2-D arrays with monolayer configuration, and these methods had been implemented in photonic devices [28-33]. In addition, the use of diblock copolymer lithography methods had enabled the fabrication of highly ordered and ultrahigh-density 2-D nanopattern arrays [34,35]. However, literatures about the influence of etching solution composition on the morphologies and properties of Si nanowire arrays are rarely reported.

In this paper, we use monocrystalline silicon wafers as the matrix, Ag as the catalyst, and hydrofluoric acid (HF) and hydrogen peroxide (H_2_O_2_) as the etching solution to prepare silicon nanowire arrays utilizing the wet chemical etching method. The photoelectric properties of the monocrystalline silicon nanowire arrays and the silicon wafers were also investigated. Additionally, in our study, we found that the increase of H_2_O_2_ concentration can influence the morphology and surface characteristics of the nanowires, which may affect their light absorption and photocatalytic properties.

## Methods

### Synthesis of SiNWs

In our experiment, (100)-oriented p-type silicon wafers were purchased and cut into 2 × 2 cm^2^ small pieces using a glass sword. A metal catalytic etching method was utilized to prepare monocrystalline silicon nanowire arrays (SiNWs). In a typical process, the pieces of the selected silicon wafers were washed by sonication in acetone and deionized water. Then, the silicon wafers were dipped into HF/H_2_O solution (1:10) to remove the thin oxidation layer and dried by N_2_ blow. Subsequently, the silicon wafers were immersed in a solution of 0.14 M HF and 0.01 M AgNO_3_ for 30 s. After a uniform layer of Ag nanoparticles was coated, the wafers were then immersed in the etchant solution composed of HF, H_2_O_2_, and H_2_O (the volume ratios are 20:10:70, 20:20:60, and 20:30:50, so the H_2_O_2_ concentration can be recorded as 10%, 20%, and 30%, respectively) at room temperature in a sealed Teflon vessel. The Si wafers were immersed in a solution of concentrated nitric acid solution to remove the excess Ag nanoparticles, rinsed with deionized water, and then dried in vacuum at 60°C.

### Characterization of SiNWs

The morphologies and microstructure of the as-synthesized SiNWs were characterized by scanning electronic microscopy (SEM; HITACHI-S4800, Chiyoda-ku, Japan) and transmission electron microscopy (TEM; JEOL JEM-2100, Akishima-shi, Japan). Ultraviolet–visible (UV–vis) absorption spectra of the SiNWs were obtained using a UV–vis spectrometer (Shimadzu UV-3600, Kyoto, Japan).

### Photoelectrochemical measurements

The photoelectrochemical measurements were carried out in a three-electrode cell in a 0.5 M Na_2_SO_4_ electrolyte solution with Si nanowire arrays, Pt electrode, and saturated mercury electrode as the working electrode, counter electrode, and reference electrode, respectively, using a CHI electrochemical analyzer (CHI 660D, CH Instruments, Chenhua Co., Shanghai, China). A 500-W xenon lamp with a light intensity of 400 mW/cm^2^ was used as the light source.

### Photocatalytic degradation of aqueous RhB over SiNWs

Photodegradation experiments were carried out in a 100-mL conical flask containing 50-mL Rhodamine B (RhB) solution with an initial concentration of 1 ppm under stirring. The prepared silicon substrate with Si nanowire arrays was put in a quartz device, and the reaction system was illuminated under a xenon lamp (light intensity of 400 mW/cm^2^). After every 1 h, 4 mL of the suspension was withdrawn throughout the experiment. The samples were analyzed using a UV–vis spectrophotometer (Shimadzu UV-3600) after removing the catalyst powders by centrifugation.

## Results and discussion

### Structure, optical properties, and photoelectric properties of SiNWs

#### SEM and TEM of SiNWs prepared with the etching solution containing 10% H_2_O_2_ (noted as 10% SiNWs)

**Figure 1:**
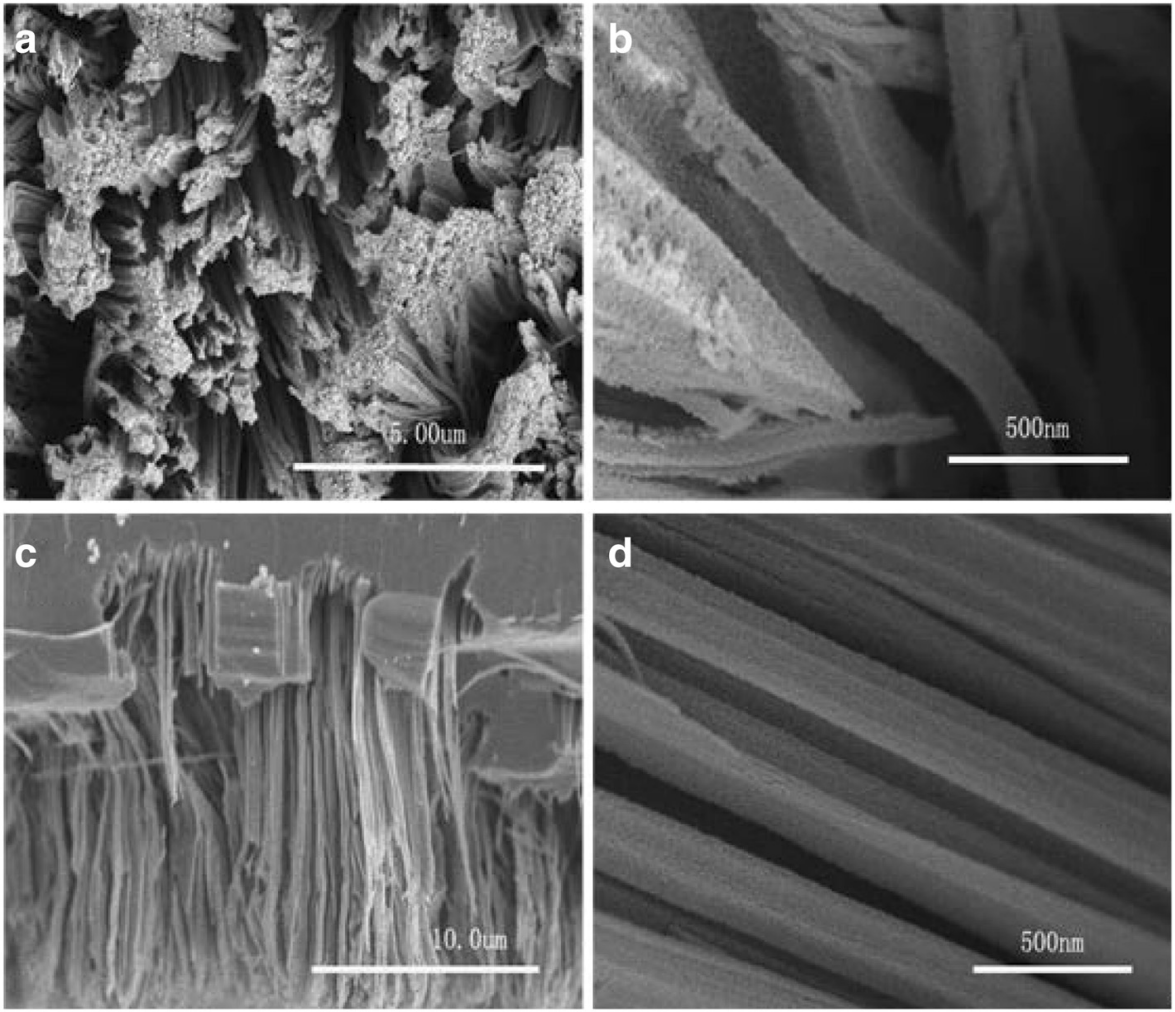
SEM images of the 10% SiNWs: (a, b) top view and (c, d) cross section.

In order to study the morphology and structure of the SiNWs, SEM and TEM measurements were performed. The SEM images of the 10% SiNWs are shown in Figure 1. From top-view images (Figure 1a,b), it can be obviously seen that SiNWs with some congregated bundles were obtained. Based on the cross-sectional SEM image (Figure 1c), the nanowires that are approximately 13 to 16 μm in length are vertical to the substrate surface. Figure 1d is the magnified cross-sectional image of the SiNWs which shows that the diameter is about 130 to 170 nm and the wires are uniform and straight. All these morphology characterizations show that through the etching reaction on silicon wafers, the Si nanowire structure has been realized. Compared with the silicon bulk material, the prepared nanowire arrays lay a reliable foundation in the structure for their improvement in photoelectric and photocatalytic performance.

**Figure 2:**
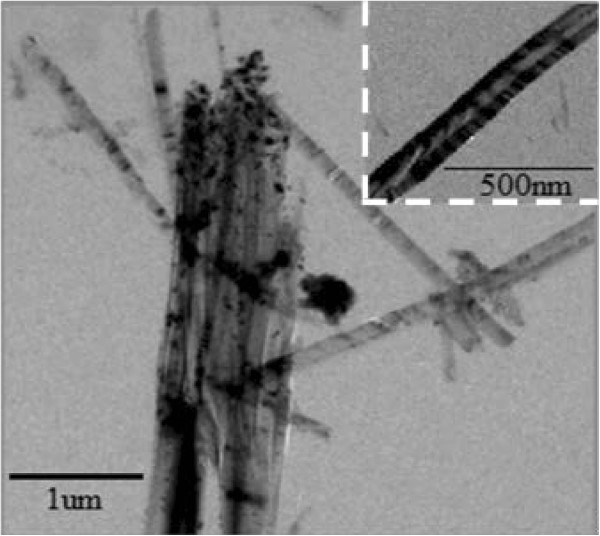
TEM image of 10% SiNWs and the high-magnification image of a selected area (inset).

Figure 2 is the TEM image of 10% SiNWs which clearly shows that the nanowires are gathered and have a bunch shape. The Si nanowires possess a diameter of about 130 to 170 nm and a length of about 3 μm, which is much shorter than that of the SEM results and may have resulted from the splitting of the silicon nanowires by ultrasonication in the sampling preparation process. The high-magnification illustration further proves that the nanowires' diameter is the same with that of the SEM test results.Moreover, it can be clearly seen that the Si nanowire displays an inhomogeneous color, indicating that the diameter of Si nanowires preared via the metal catalytic etching method is inhomogeneous.

#### UV–vis absorption and diffuse reflection spectra

**Figure 3:**
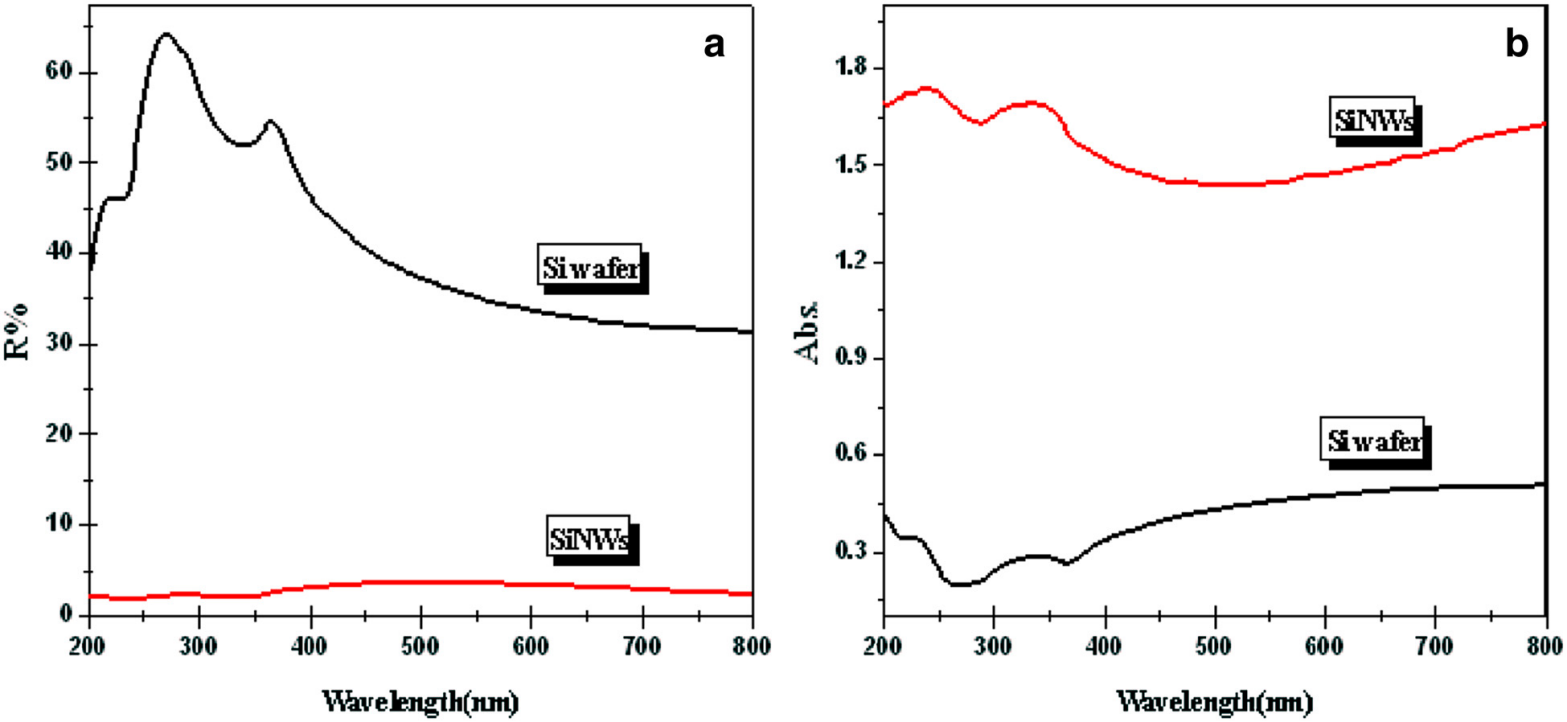
**UV–vis** (**a**) diffuse reflection and (**b**) **absorption spectra of the silicon wafer and SiNWs.**

Figure 3 compares the UV–vis absorption and diffuse reflection from a bare silicon wafer and a sample of 10% SiNWs. Figure 3a shows that the 10% SiNWs exhibit an excellent antireflection property and the reflection is below 3% for a wide range of wavelengths. It may be ascribed to the light-trapping effect caused by the construction of the SiNW nanostructure, leading to the incident light being reflected and refracted in multiple nanowire arrays and eventually being effectively absorbed. The silicon wafer shows more than 30% reflection for wavelengths 200 to 800 nm, and the reflection can be as high as 64% in ultraviolet areas. As shown in Figure 3b, the absorption spectra were converted from the reflection spectra by the standard Kubelka-Munk method, from which it can be seen that the adsorption intensity of the 10% SiNWs is obviously stronger than that of the bare Si wafer across the entire UV and visible light. The results demonstrate that the optical properties and the light absorption performance have been improved greatly due to the construction of the Si nanowire structure.

#### Photoelectrochemical results

**Figure 4:**
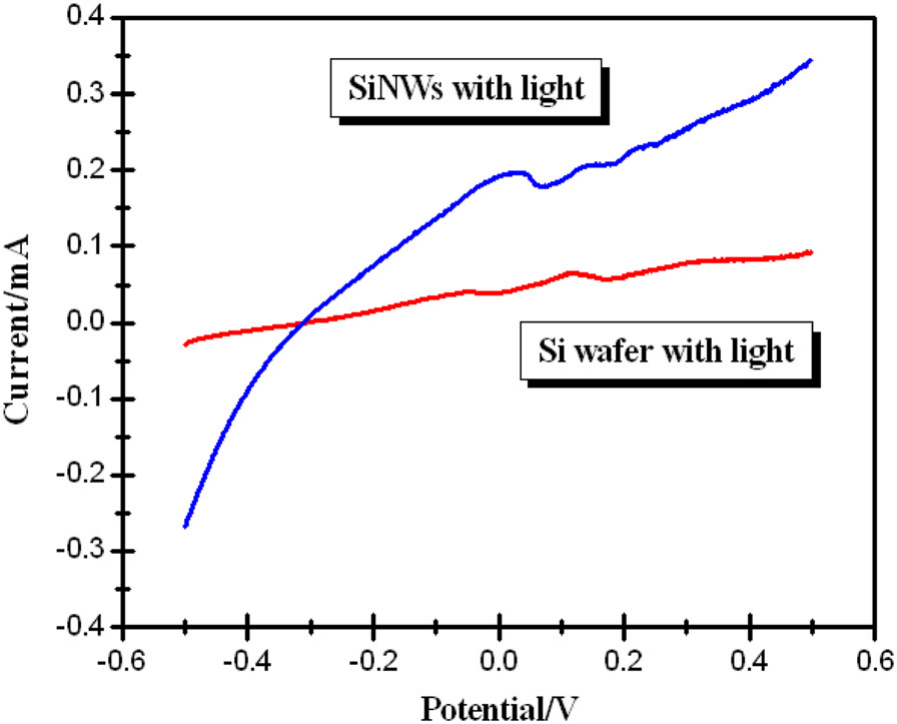
Photoelectrochemical results of silicon wafer and 10% SiNWs.

Figure 4 shows the photoelectrochemical results of the silicon wafer and 10% SiNWs. From the photoelectrochemical results of the silicon wafer and 10% SiNWs, we can obviously draw the conclusion that in the illumination condition, the light current of the 10% SiNWs is higher than that of the silicon wafer (10% SiNWs, 0.35 mA; Si, 0.09 mA; with an applied voltage of 0.5 V). The improved light current may be ascribed to the enhanced adsorption ability and photogenerated carrier separation efficiency of the 10% SiNWs, taking advantage of the formation of the Si nanowire structure. Therefore, it can be clearly inferred that the construction of the nanostructure is an effective way to improve the photoelectric performance of silicon materials.

### Influence of H_2_O_2_ concentration on the structure and photocatalytic properties of SiNWs

As H_2_O_2_ is an important component in the etching solution, our results show that the increase of H_2_O_2_ concentration can affect the morphology and surface characteristics of the nanowires. As described in the above ‘Methods’ section, we change a single-variable condition - the concentration of H_2_O_2_ in the etching process to prepare different SiNWs noted as 20% and 30% SiNWs.

#### Characterization of 20% and 30% SiNWs

**Figure 5:**
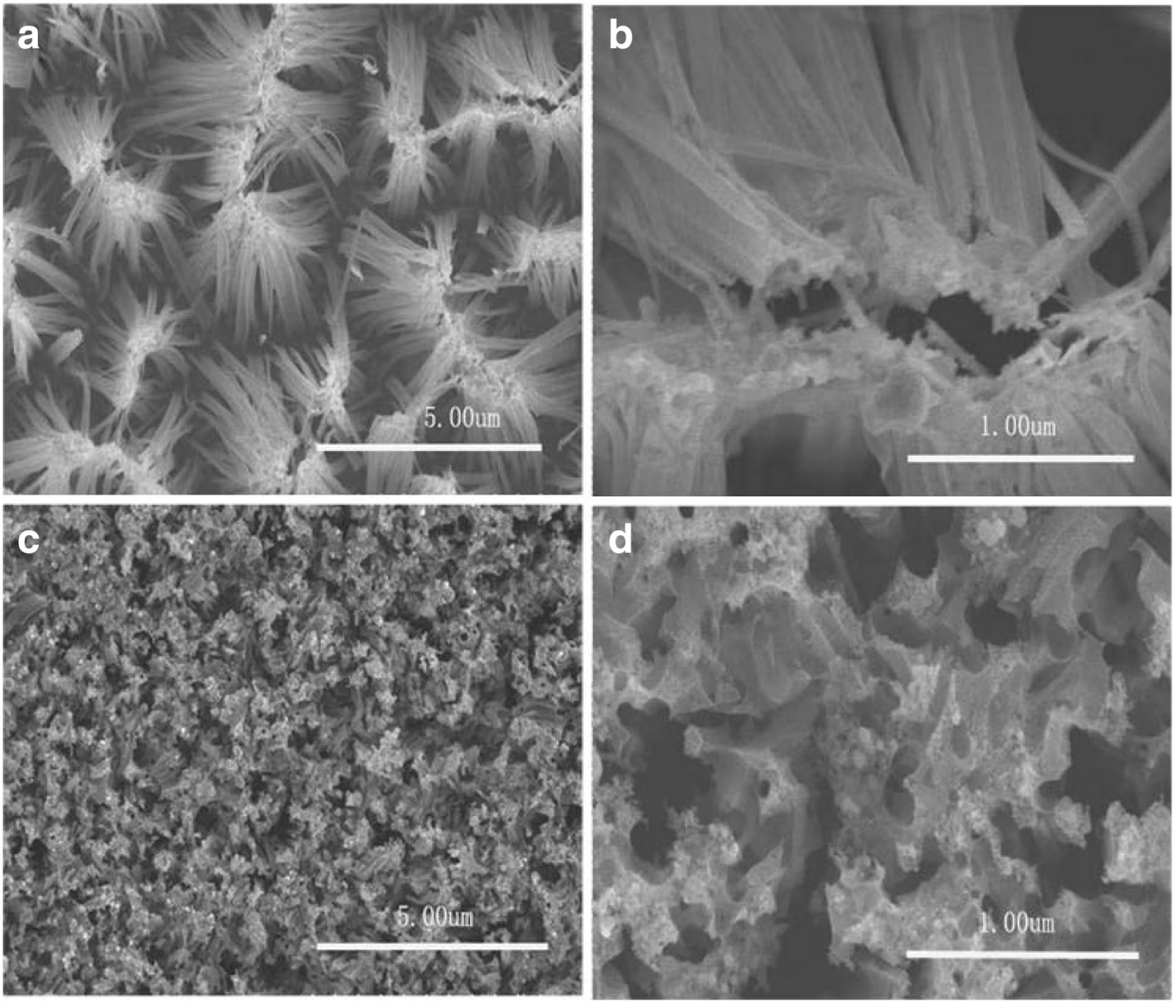
**SEM images of SiNWs with different H**_**2**_**O**_**2 **_**contents**: (**a**, **b**) **20% and** (**c**, **d**) **30%.**

Figure 5 is the SEM images of the SiNWs prepared in an etching solution with different H_2_O_2_ concentrations. It can be obviously seen from Figure 5a,b that as the concentration of H_2_O_2_ is increased from 10% to 20%, the 20% SiNWs clearly present a better linear morphology with the nanowire diameters approximately ranging from 70 to 180 nm. Moreover, in comparison with the 10% SiNWs, which show a reunion phenomenon and high nanowire density, 20% SiNWs possess a diffusion configuration and low nanowire density with the nanowire space enlarged. When the concentration of H_2_O_2_ is further increased to 30%, the prepared SiNWs do not show an expected morphology of silicon nanowire arrays but a chaotic porous structure (Figure 5c,d). With the excessive concentration of H_2_O_2_, the probability of horizontal etching increases and influences the vertical etching direction. Along with the increase of the horizontal etching speed, it may even overcome Ag particle gravity and influence of vertical etching speed and intensity, leading to a chaotic porous structure on the silicon substrate.

The morphological features above show that an appropriate improvement of the H_2_O_2_ concentration (20%) can enlarge the space of the prepared nanowires and influence their density which may affect the light absorption and photocatalytic properties. However, when the H_2_O_2_ concentration is too high (30%), a chaotic porous silicon structure, instead of nanowire arrays, is formed, caused by the horizontal etching speed overcoming Ag particle gravity and vertical etching speed under the influence of excessively high concentration of H_2_O_2_.

#### Photocatalytic activities of SiNWs

**Figure 6:**
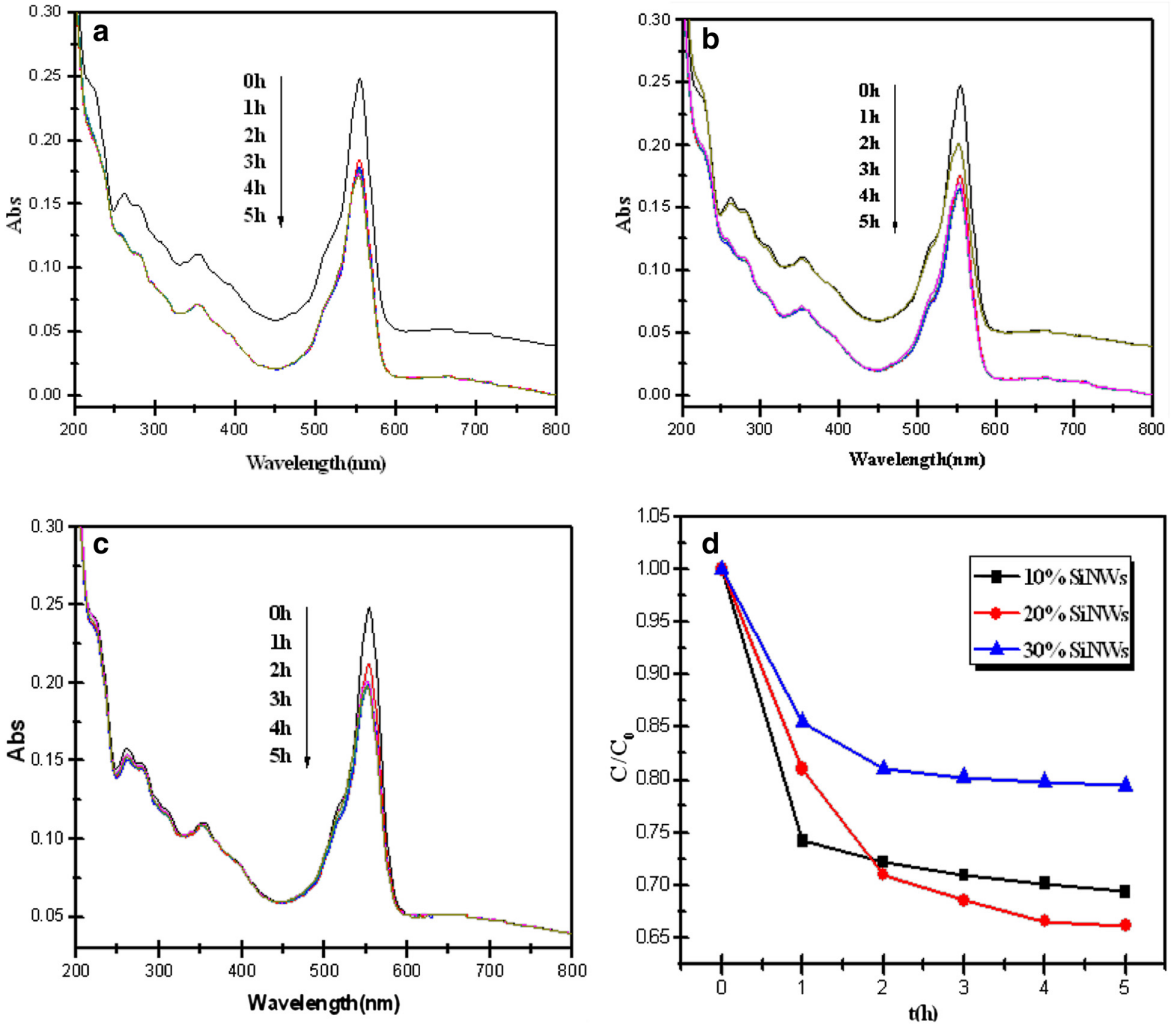
**UV**–**vis absorption spectra of RhB solution and *C***-***t *curves of SiNWs. **(**a**-**c**) UV–vis absorption spectra of RhB solution decomposed by SiNWs with different H_2_O_2_ contents under Xe arc lamp irradiation: (**a**) 10%, (**b**) 20%, (**c**) 30%. (**d**) *C*-*t* curves of the three kinds of SiNWs.

With a wide optical adsorption range and high absorption intensity, the SiNWs are expected to be potential in the photocatalytic field. A series of experiments for the photodegradation of RhB under the illumination of a 400-mW/cm^2^ xenon lamp were carried out in order to evaluate the photocatalytic activity of SiNWs (as shown in Figure 6).

As shown in Figure 6a,b,c, the typical absorption peak of RhB after degradation by 10%, 20%, and 30% SiNWs, respectively, was decreased with the extension of the irradiation time, especially in the first 1 h which may have resulted from the adsorption effect. As shown in Figure 6d, the degradation rate of RhB reached to about 30%, 35%, and 20% for 10%, 20%, and 30% SiNWs, respectively, after 5 h of irradiation. The results clearly demonstrate that the silicon nanowires can function as effective photocatalysts with light irradiation and the 20% SiNWs exhibit the highest photocatalytic decomposition efficiency, while the 30% SiNWs with a chaotic porous structure was the worst. The enhanced catalytic activity of the 20% SiNWs could be attributed to their morphology characterization which possesses an appropriate nanowire density to optimize the effect of Si content and contact area of the photocatalyst and RhB.

### Formation mechanism of SiNW arrays

In brief, the metal-assisted chemical etching method to prepare silicon nanowires is a process in which silicon is oxidized into SiO_2_ using metal nanoparticles (such as Au, Ag, Fe, etc.) as catalysts and H_2_O_2_ as oxidant and then etched using HF solution.

**Figure 7:**
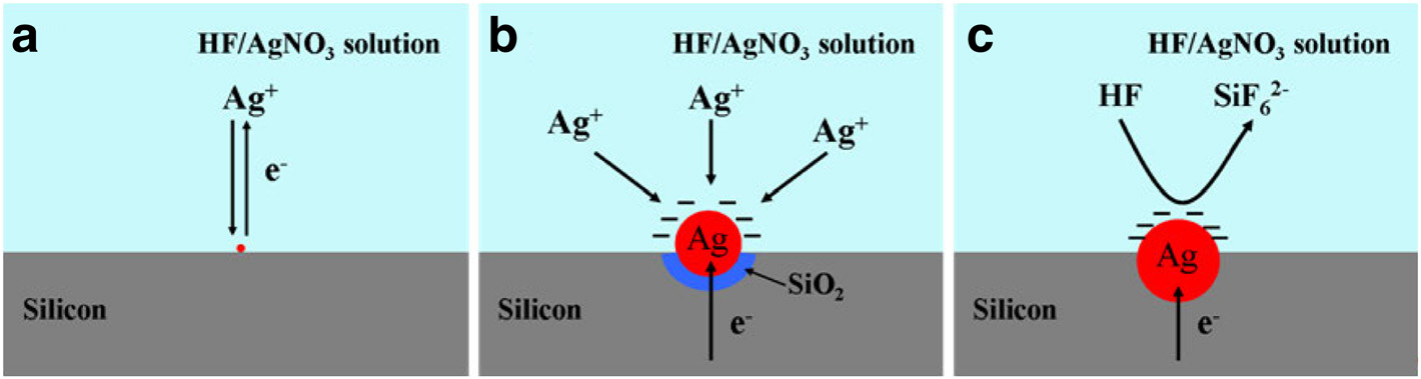
**Mechanism diagram of Ag deposition on the Si surface in HF**/**AgNO**_**3 **_**solution.** (**a**) Formation of Ag nucleation. (**b**) Ag particle growth and Si substrate oxidation. (**c**) Ag particles trapped in the pits formed by the etching of SiO_2_ around it by HF.

Metal-assisted chemical etching to prepare silicon nanowires can be divided into two processes (taking Ag as an example):

1. As shown in Figure 7a, when the silicon wafer is immersed into AgNO_3_/HF mixture solution, silver ions in the vicinity of the silicon surface capture electrons from silicon and deposit on the silicon substrate surface in the form of metallic silver nuclei; at the same time, the silicon around the silver nuclei is oxidized to SiO_2_. The process is the same as the mechanism of the deposition of copper nanoparticles on silicon substrate surface [36], which is the replacement reaction, and can be divided into two synchronous reaction steps (the cathode reaction and the anode reaction):

a. Cathode reaction:

Ag^+^ + e^−^ = Ag*E*^*θ*^ = 0.79 V

b. Anode reaction:

Si + 2H_2_O = SiO_2_ + 4H^+^ + 4e^−^*E*^*θ*^ = 0.91 V

SiO_2_ + 6HF = SiF_6_^2−^ + 2H_2_O + 2H^+^

c. Overall reaction:

Si + 6HF + 4Ag^+^ = 4Ag + SiF_6_^2−^ + 6H^+^

The silver nuclei attached to the Si substrate have higher electronic activity than silicon atoms and constantly obtain electrons from silicon atoms, which makes the cathode reaction to occur constantly and results in the silver nuclei gradually growing up to form silver nanoparticles (as shown in Figure 7b). At the same time, the silicon atom around the silver nanoparticles is oxidized to SiO_2_ and dissolved by HF in the form of SiF_6_^2−^, leading to the Ag nanoparticles down into the wafer (Figure 7c).

**Figure 8:**
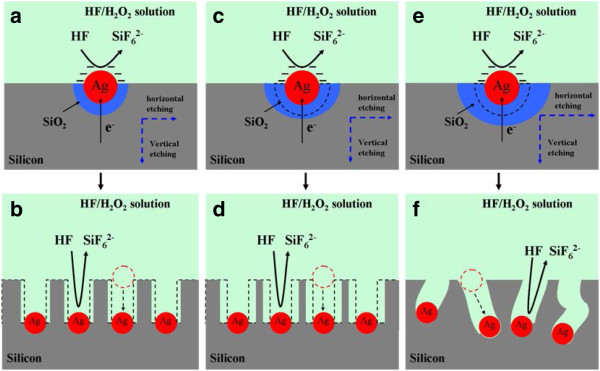
**Schematic diagram of Ag nanoparticle**-**assisted etching with the increase of H**_**2**_**O**_**2 **_**concentration: (a, b) 10%, (c, d) 20%, and (e, f) 30%.**

2. As shown in Figure 8a, when the silicon substrate deposited with silver nanoparticles is immersed in HF-H_2_O_2_ etching solution, SiO_2_ is continuously formed from the silicon contacted with silver nanoparticles with H_2_O_2_ as hole donor and oxidant and dissolved by HF, leading to the sinking of the silver grains. With the silicon around the silver nanoparticles constantly oxidized and dissolved, the silicon substrate is etched to form silicon nanowires (Figure 8b):

a. Cathode reaction:

H_2_O_2_ + 2H^+^ → 2H_2_O + 2 h^+^*E*^*θ*^ = 1.76 V

b. Anode reaction:

Si + 6HF + nh^+^ → H_2_SiF_6_ + nH^+^ + [n / 2]H_2_

c. Overall reaction:

Si + 6HF + n / 2H_2_O_2_ → H_2_SiF_6_ + nH_2_O + [2 − n / 2]H_2_

In the process, AgNO_3_ plays an important role in forming silver grains as a catalyst to promote the etching reaction. Previous research [37] shows that in metal auxiliary etching, the formation of vertical nanowires is relative to etching limitation around silver nanoparticles. Silver nanoparticles on silicon surface could catalyze the etching reaction around and below the silicon substrate to form pits and then sink into the pits as a result of gravity, so the etching reaction is along the vertical direction.

With the increase of H_2_O_2_ concentration which acts as hole donor and oxidant in the etching process, the oxidation speed of the silicon around the Ag nanoparticles increases, resulting in the increase of the horizontal etching speed of the silicon. When the H_2_O_2_ concentration reaches 20% in the etching solution, as shown in Figure 8c, more silicon around Ag nanoparticles will be oxidated into SiO_2_ and then dissolved by HF, leading to an increased horizontal etching speed, which results in the 20% SiNWs possessing a diffusion configuration and low nanowire density with the nanowires space enlarged (Figure 8d). When the concentration of H_2_O_2_ is further increased to 30%, the horizontal etching speed increases in a higher degree and overcomes the Ag nanoparticle gravity to shift its position, deviating from the vertical direction (Figure 8e). Finally, the prepared SiNWs do not present an expected morphology of silicon nanowire arrays but a chaotic porous structure on the silicon substrate (Figure 8f).

## Conclusions

SiNWs have been prepared successfully through a simple, convenient, and controllable metal-assisted chemical etching method. The formation mechanisms, electrical properties, and optical properties as well as photocatalytic performances have also been studied. The photoelectrochemical results show that the formation of the Si nanowire structure greatly improved the photoelectric performances. By changing the H_2_O_2_ concentration in the etching solution, we get 10%, 20%, and 30% SiNWs with different morphologies of high-density nanowire arrays, low-density nanowire arrays, and a chaotic porous nanostructure, respectively. The photocatalytic research shows that 20% SiNWs exhibit an enhanced photocatalytic activity than 10% and 30% SiNWs, which could be ascribed to the appropriate nanowire density with the effect of Si content and contact area of photocatalyst and RhB optimized.

## Competing interests

The authors declare that they have no competing interests.

## Authors’ contributions

YL carried out the preparation and main characterization of the SiNWs, participated in the sequence alignment, and drafted the manuscript. GJ carried out the performance test and participated in its design and coordination. JW participated in the data analysis and English description modification. XL participated in the UV–vis spectra testing and analysis. ZZ participated in the formation mechanism analysis of SiNWs. YS participated in the design of the study. All authors read and approved the final manuscript.
